# Oral lesions in human monkeypox disease and their management

**DOI:** 10.1002/cre2.690

**Published:** 2023-05-05

**Authors:** Betsy Joseph, Sukumaran Anil

**Affiliations:** ^1^ Department of Periodontics, Saveetha Dental College and Hospital Saveetha Institute of Medical and Technical Sciences Chennai India; ^2^ Department of Dentistry, Oral Health Institute Hamad Medical Corporation Doha Qatar; ^3^ College of Dental Medicine Qatar University Doha Qatar

**Keywords:** disease management, Monkeypox, Monkeypox virus, oral manifestations, oral ulcer

## Abstract

**Objective**: The current outbreak of human monekypox (MPX) in several endemic and non‐endemic regions in 2022 has generated significant international attention. Despite the early classification as zoonotic, MPXV has demonstrated the potential for human‐to‐human transmission through close contact with lesions, body fluids, respiratory droplets, and contaminated materials. Therefore, our objective was to elaborate on the oral lesions in human MPX and their management.**Materials and Method**: Articles published up to August, 2022, were screened to identify relevant studies in humans that reported oral lesions in MPX.**Results**: Oral lesions have been found to manifest differently and transform from vesicles to pustules, accompanied by umbilication and crusting within four weeks. Along with fever and lymphadenopathy, these lesions may develop in the oral cavity and then spread to the skin surrounding the extremities in a centrifugal pattern. In some patients, the oropharyngeal and perioral lesions were the initial presentations.**Conclusions**: The oral lesions of MPX infection and its management strategies are relevant for dentists. Dental practitioners may be the first to detect the initial lesions of MPX. Therefore, high alertness should be there, especially while examining patients with fever and lymphadenopathy. It is also essential to thoroughly examine the oral cavity for macular and papular lesions in oral mucosa, tongue, gingiva, and epiglottis. Symptomatic and supportive care of oral lesions is recommended.

## 1 INTRODUCTION

Monkeypox (MPX) is a viral disease of animal origin (zoonosis) caused by a Monkeypox virus (MPXV) virus of the orthopox family and is closely associated with smallpox viruses. The current outbreak of MPX in several endemic and non‐endemic regions in 2022 has generated significant international attention. This multicountry outbreak is caused by consistent human‐to‐human transmission among nontravelers, primarily men who engage in sexual activity with other men (MSM). As of August 9, 2022, there are around 30,000 confirmed cases of MPX spreading in 93 nonendemic counties (WHO, 2022). Despite the early classification as zoonotic, the MPXV has demonstrated the potential for human‐to‐human transmission through close contact with lesions, body fluids, respiratory droplets, and contaminated materials. In addition, its sexual transmission would be a cause for concern. The MPXV was also detected in the patients' seminal fluid, genital and rectal lesions, and feces (Adler et al., 2022; Jang et al., 2022).

The clinical diagnosis of MPX requires laboratory confirmation, and a swab is collected from the lesion exudate or crust to isolate viral nucleic acids for diagnosis (Thornhill et al., 2022; WHO, 2022). The MPXV genome‐specific polymerase chain reaction (reverse transcription‐polymerase chain reaction) using the viral DNA is used to confirm the diagnosis (WHO, 2022). The differential diagnosis of MPX, including smallpox, measles, chickenpox, syphilis, and others, depends on the local epidemiology, such as Peruvian warts in various places in Latin America or Buruli ulcer in Africa.

The clinical manifestations of MPX include nonspecific symptoms such as fever, chills, myalgia, headache, lethargy, and lymphadenopathy, followed by a vesiculo‐pustular rash with an incubation period ranging from 5 to 21 days (Thornhill et al., 2022). Ulcers in the oral cavity or oropharynx can be the primary lesions (Ajmera et al., 2022; Tarín‐Vicente et al., 2022) and then spread to the face and extremities, including the palms and soles. Each lesion begins as a macule and then progresses to papules, vesicles, pustules, and scabs (WHO, 2022). In the current outbreak, perioral papules have been reported that blistered and ulcerated (Ajmera et al., 2022).

## 2 ORAL LESIONS

The oral ulcerations and management of the cases reported recently are outlined in Table 1. It was discovered that nearly a quarter (23.5%) of the people with MPXV had mouth ulcers. Oral lesions have been found to manifest differently and transform from vesicles to pustules, accompanied by umbilication and crusting within 4 weeks (Ajmera et al., 2022; Thornhill et al., 2022). Along with fever and lymphadenopathy, these lesions may develop in the oral cavity and then spread to the skin surrounding the extremities in a centrifugal pattern. In some patients, the oropharyngeal and perioral lesions were the initial presentations (Thornhill et al., 2022). Oral Candidiasis was also presented in cases (Ajmera et al., 2022). Even in the absence of skin lesions in the prodromal stage, viral DNA particles were detected in patients with oral lesions' oral and pharyngeal passages. Regardless of the route of infection, oral secretions revealed a high virus concentration in animal experiments (Ajmera et al., 2022; Tarín‐Vicente et al., 2022). Oral lesions on the oral epithelium, tongue, and labial mucosa of infected sites exhibited epithelial hyperplasia, intracellular edema, necrosis, ulceration, and scattered and poorly defined eosinophilic intracytoplasmic inclusion bodies. The tongue's stratified squamous epithelium contained multinucleated syncytial cells. During immunohistochemistry analysis, orthopoxvirus antigens were exclusively detected in the skin and oral cavity.

**Table 1:** Oral lesions in the current human monkeypox and their management

Author	Oral lesions/management
Ajmera et al. (2022)	One case reported oral lesions, tongue swelling, and a burning sensation in his mouth. Multiple umbilicated perioral and oral lesions and oral candidiasis were found. The patient was given magic mouthwash, IV fluconazole for oral thrush, an intramuscular dose of penicillin, and supportive care.
Thornhill et al. (2022)	Oral lesions in 13/30 cases (total 528 patients). Within 7 days, ulcers on the tongue, corner of the mouth, and perioral umbilicated and vesicular lesions. Antiviral treatment.
Patel et al. (2022)	27/197 patients had oral lesions. No specific management for oral lesions.
Girometti et al. (2022)	4/54 had an oropharyngeal lesion. No specific management for oral lesions.
Matias et al. (2022)	Three case reports. Painful, pruritic vesiculo‐pustular oropharyngeal lesions/pustular lesions on his gingiva. Tecovirimat 600 mg twice daily orally for 14 weeks for systemic lesions.
Tarin Tarín‐Vicente et al. (2022)	78/181 cases had lesions in the oral and perioral region. No specific management mentioned.
Jang et al. (2022)	In one case report, perioral erosive lesions covered with black crusts were found on his face. No specific management mentioned.
Peiró‐Mestres et al. (2022)	Oral and pharyngeal lesions in 12 patients. No specific management mentioned.

## 3 MANAGEMENT OF ORAL LESIONS

Most reports do not mention specific management of oral lesions. Although the MPX disease is self‐limiting, prompt management is essential. Symptomatic treatment is the mainstay of therapy, especially during the prodromal phase. The interim rapid response guidance released on June 10, 2022, by WHO recommends rinsing the mouth with salt water at least four times a day in case of oral lesions (WHO, 2022). The oral lesions can source secondary infection and facilitate person‐to‐person viral transmission. A clean, moist microenvironment can mitigate transmission potential by covering infectious sores and promoting the re‐epithelization of the damaged exanthem (WHO, 2022). Using an oral antiseptic solution such as chlorhexidine mouthwash can keep the lesions clean. Alternatively, cleansing the ulcers with a dilute povidone‐iodine solution will help maintain a hygienic microenvironment. Topical application of anesthetics such as viscous lidocaine is used for symptomatic relief (WHO, 2022). Systemic and topical analgesics are used to control the pain of oral mucosal lesions. Patients with secondary bacterial infections should be treated with appropriate antibiotics. The use of nonsteroidal anti‐inflammatory drugs (NSAIDs) for pain relief should be limited due to the concern of developing hemorrhagic lesions. “Magic mouthwash,” along with intravenous fluconazole for oral candidiasis and an intramuscular dose of penicillin, demonstrated symptomatic relief in patients (Ajmera et al., 2022).

Oral acyclovir may be prescribed at a prophylactic dose of 50 mg/kg given twice daily. Many oropharyngeal lesions, such as tonsillar edema and odynophagia, can be relieved within 5 days when tecovirimat is administered for skin lesions (Matias et al., 2022). Cidofovir, Brincidofovir, and Tecovirimat are the antivirals used in MPX and as a postexposure prophylactic (PEP) agent in exposed immunocompromised individuals who are contraindicated to receiving the smallpox vaccine as a PEP measure (Matias et al., 2022). Patients with oral lesions should be monitored for dehydration and malnutrition and maintain good nutrition and hydration. Complications of illness include low mood, and emotional lability may expose the patients to poor oral health practices (Adler et al., 2022).

The oral lesions of MPX infection and its management strategies are relevant for dentists. Dental practitioners may be the first to detect the initial lesions of MPX. Therefore, high alertness should be there, especially while examining patients with fever and lymphadenopathy. It is also essential to thoroughly examine the oral cavity for macular and papular lesions in the oral mucosa, tongue, gingiva, and epiglottis. Symptomatic and supportive care of oral lesions is recommended.

## AUTHOR CONTRIBUTIONS

**Betsy Joseph** and **Sukumaran Anil**: Conceptualization; writing ‐ original draft; writing ‐ review, and editing.

## CONFLICT OF INTEREST

The authors declare no conflict of interest.

## Data Availability

NA.
